# Extracellular vesicle-mediated heterogeneous communication between cancer and the lymphatic system facilitates lymphatic metastasis

**DOI:** 10.20892/j.issn.2095-3941.2023.0007

**Published:** 2023-03-24

**Authors:** Changhao Chen, Yuming Luo, Hanhao Zheng, Dingwen Zhang, Yao Kong, Jiabin Yang, Mingjie An, Yan Lin, Daowei Lin, Rufu Chen

**Affiliations:** 1 Department of Urology, Sun Yat-sen Memorial Hospital, Sun Yat-sen University, Guangzhou 510120, Guangdong, China; 2 Guangdong Provincial Key Laboratory of Malignant Tumor Epigenetics and Gene Regulation, Sun Yat-sen Memorial Hospital, State Key Laboratory of Oncology in South China, Guangzhou 510120, Guangdong, China; 3 Department of Pancreatic Surgery, Guangdong Provincial People’s Hospital, Guangdong Academy of Medical Sciences, Guangzhou 510080, Guangdong, China; 4 Department of Anesthesiology, Sun Yat-sen Memorial Hospital, Sun Yat-sen University, Guangzhou 510120, Guangdong, China

## Introduction

The dissemination of cancer cells to organs initiates the formation of an aggressive cancer phenotype and is a predominant cause of cancer-associated death. For most epithelial cancers, lymphatic system metastasis has been characterized as the most common and earliest metastatic pathway, and the detection of metastasis in lymph nodes (LNs) often predicts poor survival among patients^1^. Although increasing attention is being paid to the clinical importance of LN metastasis, the underlying mechanisms have remained unclear in the past decade. Accumulating evidence suggests that the occurrence of LN metastasis is not stochastic but is a programming biological event regulated by the bidirectional crosstalk between metastasis-initiating cancer cells and the tumor microenvironment (TME)^2^. However, the regulators and patterns of cancer-TME communication in LN metastasis remain to be further explored.

Extracellular vesicles (EVs) are phospholipid bilayer vesicles that are classified into exosomes (30–150 nm) and microvesicles (50–1,000 nm) according to their source and size. EVs have important intercellular communication functions that facilitate a variety of pathological processes in human diseases, particularly in cancers^3^. EV-mediated communication facilitates signal transduction between cancer cells and the TME, thereby enabling the formation of a pre-metastatic niche and conditions favoring tumor LN metastasis^4^. Thus, investigation of the mechanism underlying EV-mediated LN metastasis is an emerging area of research. Targeting EVs may hold promise in the treatment of LN metastatic caners.

## Mechanisms of EV-mediated lymphangiogenesis

LN metastasis is a complicated multistep process comprising intratumoral and peritumoral lymphangiogenesis, detachment from the original tumor into the matrix, anchoring of tumor cells to lymphatic vessels, and colonization of hospitable LNs. Among these steps, lymphangiogenesis, the formation of new lymphatic vessels from pre-existing lymphatic vessels, is considered rate-limiting in LN metastasis^1^. Previous studies have demonstrated that, unlike vascular endothelial cells (ECs), lymphatic ECs (LECs) do not have a basement membrane, are rarely covered by smooth muscle, and are not continuously connected. PROX1 is the main regulatory factor driving lymphatic differentiation^1^. In addition, capillary lymphatic epithelial cells often express hyaluronic acid receptor 1 (LYVE-1), p protein, and vascular endothelial growth factor receptor 3 (VEGFR3), which characterize the main phenotypes of LECs^1^. Differ from lymphatic epithelial cells, the specific marker of vascular ECs is CD31. Lymphangiogenesis has been proposed to be driven by 2 mechanisms: VEGF-dependent and VEGF-independent regulation.

### Roles of EVs in VEGF-dependent lymphangiogenesis

**Figure 1:**
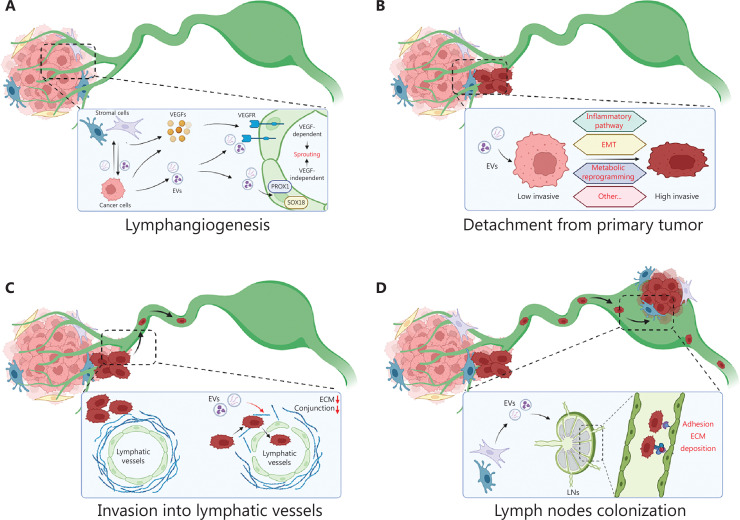
Mechanisms of EVs in regulating different steps of LN metastasis. Proposed models for: (A) the role of EVs in mediating lymphangiogenesis; (B) the role of EVs in inducing the aggressive phenotype of cancer cells and leading to their detachment from primary tumors; (C) the role of EVs in destroying the EC barrier of lymphatic vessels and fostering the invasion of cancer cells into lymphatic vessels; and (D) the role of EVs in triggering cancer cells to colonize LNs.

VEGF protein family members, including VEGF-A, VEGF-B, VEGF-C, VEGF-D, VEGF-E, and placental growth factor, contribute to the differentiation of ECs^1^. VEGF-A and VEGF-B interact predominantly with VEGFR1 and VEGFR2 expressed on vascular ECs, and mediate proliferation and tube formation in vascular ECs rather than LECs. VEGF-C, the predominant VEGF family ligand for VEGFR3 located on LEC membranes, plays a crucial role in tumor-associated lymphangiogenesis^5^. Studies have confirmed that VEGF-C signaling is regulated by a variety of molecules and pathways in cancers, and EVs have been widely reported to be involved in the activation of cancer cell-secreted VEGF-C. Glioblastoma-derived EVs activate the VEGF-C/VEGFR2 signaling pathway through the delivery of VEGF-C, thus eventually promoting LEC viability and tube formation^5^. EV-packaged BCYRN1 secreted by bladder cancer cells epigenetically increases WNT5A expression, thereby facilitating VEGF-C secretion, and forms an WNT5A/VEGF-C/VEGFR3 feedforward loop that enhances lymphangiogenesis^6^. Beyond the crucial role of EVs in cancer cell-derived VEGF-C secretion, EVs participate in triggering VEGF-C secretion by the cellular components, such as microphages and fibroblasts, in TME. CCL2-decorated EVs directly targeted CCR2 receptor expressed on the membrane of tumor associated macrophages and promotes lymphangiogenesis through inducing VEGF-C section by tumor associated macrophages^3,7^. Thus, EV-mediated VEGF-C-associated lymphangiogenesis is achieved through dual regulation of both cancer cells and the TME (**Figure 1A**).

VEGF-D, another essential member of the VEGF family, serves as a homolog of VEGF-C and participates in the induction of physiologic lymphangiogenesis. In recent studies, the upregulation of VEGF-D has been reported in several cancers and has been found to be accompanied by LN metastasis^8^. These findings suggest that VEGF-D might also regulate tumor lymphangiogenesis during LN metastasis. Similarly to VEGF-C, VEGF-D directly binds VEGFR3 receptors on LECs and activates the expression of lymphangiogenesis-associated genes, thereby inducing the sprouting of lymphatic vessels^8^. Few studies have reported the regulation of VEGF-D in cancers, most of which have focused on the roles of intrinsic transcriptional factors or noncoding RNAs^8^ in facilitating VEGF-D secretion. However, VEGF-D has been detected in EVs, and its secretion is activated after EV induction. Therefore, EVs may also play an important regulatory role in VEGF-D-associated tumor lymphangiogenesis, but the role and mechanisms must be further elucidated (**Figure 1A**).

### Roles of EVs in VEGF-independent lymphangiogenesis

Although VEGFs substantially contribute to tumor lymphangiogenesis, some patients with LN metastasis have low VEGF expression, thereby indicating the existence of VEGF-independent lymphangiogenesis^9^. Studies have demonstrated that lymphangiogenesis begins with the expression of PROX1 in ECs, thus facilitating the detachment of ECs from the embryonic cardinal vein wall^1^. Consequently, direct regulation of PROX1 and SOX18 expression is a major pattern in VEGF-independent lymphangiogenesis. Beyond VEGF pathways, EVs directly affect the expression of key regulators of lymphatic vasculature in LECs. EV-packaged CD44 promotes PROX1 expression *via* the YAP/CPT1A axis and induces lymphangiogenesis in gastric cancer^10^. Bladder cancer-derived EVs are internalized by LECs and subsequently induce the transcription of SOX18 and PROX1 through the transmission of ELNAT1 and LNMAT2, thereby facilitating lymphangiogenesis in a VEGF-independent manner^9,11^. In our center, we have focused on the production of EVs during VEGF-independent lymphangiogenesis and have found that SUMOylation is an essential event triggering the encapsulation of EVs. Moreover, KRAS mutation has been identified as a trigger for the SUMOylation of key components of EVs, thus promoting the assembly of endosomal sorting complex required for transport (ESCRT), and the targeting of PROX1 or SOX18 in LECs^12^. Because most epithelial cancers are characterized by SUMOylation or KRAS mutation, the abovementioned researches might provide a promising intervention strategy for LN metastasis if similar mechanisms exist in other cancers (**Figure 1A**).

## Mechanisms underlying aggressive EV-mediated phenotypes in cancer cells

An increase in the invasiveness and malignant proliferation of cancer cells causes their detachment from primary tumors and infiltration into peritumoral regions enriched in lymphatic vessels, thus leading to LN metastasis in various malignancies^13^. The invasion of sentinel LNs often indicates that tumor cells have broken through the lymphatic vessel barrier and achieved distant metastasis. Previous studies have demonstrated that EV-mediated transport of various functional molecules, such as oligopeptides, mRNAs, non-coding RNAs, and metabolites, to cancer cells endows these cells with an invasive phenotype by affecting their characteristics^3^. Epithelial-mesenchymal transition (EMT) has been well characterized as an indispensable process in the induction of cancer cell plasticity and promotion of metastasis^10^. CD103-positive EVs derived from cancer stem cells transport miR-19b-3p, which in turn initiates EMT and confers high metastatic capacity on cancer cells. Moreover, the aberrant activation of inflammatory signaling pathways, such as the NF-κB or TGF-β pathways, is closely correlated with the invasive ability of cancer cells^13^. Cancer-associated fibroblast-derived EVs deliver miR-17-5p to cancer cells, thereby activating the TGF-β signaling pathway and forming a positive feedback loop facilitating metastasis. EV-packaged miR-1910-3p downregulates myotubularin-associated protein 3, and subsequently activates the NF-κB signaling pathway and promotes breast cancer metastasis. Furthermore, reprogramming of lipid metabolism causes the migration of cancer cells into the lipid-enriched lymphatic system, thereby contributing to the invasion of lymphatic vessels^10^. Adipose-derived EVs regulate lipid metabolism by delivering MTTP into cancer cells, which impaired polyunsaturated fatty acid ratio. Beige/brown adipocyte differentiation and metabolic remodeling induced by EV-packaged miR-144 and miR-126 result in metabolic adaptation and an aggressive phenotype of cancer cells. Therefore, EVs have a variety of molecular mechanisms and biological functions in regulating the invasiveness of cancer cells during LN metastasis (**Figure 1B**).

## Other mechanisms of EV-mediated LN metastasis

Beyond promoting lymphangiogenesis and the invasiveness of tumor cells, EVs are widely involved in other process of LN metastasis. Tumor cell derived EVs destroy the tight junctions of ECs by decreasing ZO1 and increasing the permeability of the lymphatic vessel barrier, thereby facilitating tumor cell entry into lymphatic vessels. Thus, EVs appear to have crucial roles in the destruction of the EC barrier of lymphatic vessels and the invasion of cancer cells into neonatal lymphatic vessels^10^ (**Figure 1C**). LECs are regulated by tumor-derived EVs, thus resulting in LEC dysfunction and increased permeability of lymphatic vessels. miR-1468-5p in tumor secreted EVs reprograms LECs *via* the HMBOX1-SOCS1-JAK2/STAT3 axis, thus increasing the permeability of the lymphatic vessel barrier and promoting lymphatic metastasis. Moreover, lymphoid circulation is enriched by variety of immune cells, which are highly lethal toward tumor cells. The immune escape induced by tumor derived EVs plays a crucial role in LN metastasis. EV-delivered TGF-β1 significantly induces the regulatory T (Treg) cells infiltrating into celiac LNs, thus providing an immunosuppressive microenvironment supporting lymphatic invasion by cancer cells. Furthermore, EVs have multiple functions in fostering the colonization of cancer cells in LNs. Primary tumors secrete EVs as systematic signals resulting in remodeling of the extracellular matrix (ECM) structure in draining LNs, and the formation of a pre-metastatic niche allowing cancer cells to survive and metastasize^14^. Melanoma-derived EVs have been reported to drain into sentinel LNs *via* lymphatic trafficking, thereby triggering ECM deposition and vascular proliferation in LNs^14^. Notably, the chemokine receptor CCR7 and its ligands CCL19 and CCL21 control multiple migration events in adaptive immune function. Studies have shown that EV-packaged CCR7-induced tumor cells recognizes and accumulates toward lymphogenic CCL19 and CCL21, thereby promoting preferential tumor cell localization in sentinel LNs^1^. However, EV-packaged ICAM-1 and MMP-9 induce vascular metastasis of tumor cells^15^. In contrast to tumor-derived EVs, stromal cell derived EVs modify the dormancy and outgrowth of disseminated cancer cells, thus favoring the colonization and survival of cancer cells^16^ (**Figure 1D**). Together, tumor derived EVs are involved in the regulation of LN metastasis through multi-dimensional molecular mechanisms, thereby supporting their essential roles in promoting LN metastasis.

## Clinical relevance of EVs in LN metastasis

In the past decade, molecules including mRNAs, double-stranded DNA fragments, lipids, and lncRNAs in EVs have received substantial attention as potential biomarkers and therapeutic targets for multiple cancers^17^.

Next generation sequencing of serum EVs from patients has confirmed the diagnostic value of tumor-derived EV lncRNAs in cervical squamous cell carcinoma, and miR-221-3p has been identified as a diagnostic marker of LN metastatic cervical squamous cell carcinoma. Moreover, the assessment of LNMAT2^9^ and ELNAT1 expression in urinary EVs has been found to predict LN metastasis in bladder cancer^11^. These findings provide a reference for the development of highly sensitive and non-invasive diagnostic kits.

In addition, EV-mediated interactions between tumor cells and LECs in KRAS^G12D^ pancreatic cancer create conditions supporting lymphangiogenesis and LN metastasis, and inhibiting the expression of EV-packaged hnRNPA1 has shown satisfactory effects in the inhibition of LN metastasis *in vitro* and *in vivo*^12^. Moreover, bladder cancer-derived EVs promote lymphangiogenesis and LN metastasis through ELNAT1 delivery. In animal models, specifically blocking EV-mediated ELNAT1 delivery effectively inhibits LN metastasis in bladder cancer^11^. These findings reveal the potential for use of EV-packaged molecules as therapeutic targets in multiple cancers.

## Discussion and perspectives

Because LN metastasis occurs frequently and leads to poor outcomes in patients with cancer, developing feasible approaches to monitor and target LN metastasis remains urgently needed. In recent years, EV-packaged molecules have been shown to have high stability and abundance, and specific expression profiles in LN metastatic diseases^4^. Moreover, most EVs have been demonstrated to act as crucial regulators of lymphangiogenesis, aggressive phenotype formation and LN colonization during LN metastasis^1^. Owing to the properties and functions of EV-packaged molecules, developing EV-based biomarkers and therapeutic strategies might provide novel insights into diagnosis and treatment for LN metastasis.

Current strategies for diagnosing LN metastasis before surgery or biopsy rely on imaging-based methods, including CT, MR, or echoendoscopy, which have fairly low accuracy for multiple cancers. Recently, EVs have been shown to have potential as biomarkers for identifying metastatic tumors^17^. EV-delivered molecules, such as lncRNAs, miRNAs, circRNA or proteins, have been identified in the body fluids of patients with LN metastatic cancers, and have shown satisfactory performance in assessing LN status. EVs containing melanoma-associated proteins and miRNAs exhibit unique signatures differentiating early stages of LN metastasis, and may potentially be applied in therapies to halt metastatic spread^4^. Moreover, approximately 60% of missed-diagnosed LN status using imaging-based methods were correctly predicted to be LN-positive by the detection of the EV-packaged ELNAT1 in bladder cancer, and thus might serve as an alternative for the diagnosis of LN metastasis^11^. Our recent study has also indicated that pancreatic cancer with KRAS^G12D^ mutation is accompanied by elevated incidence of LN metastasis. High-throughput sequencing has been applied to identify the molecules specifically expressed in KRAS^G12D^ pancreatic cancer, and the clinicopathologic features have been further analyzed. HnRNPA1 has been identified as a specific molecular marker of serum EVs in patients with KRAS^G12D^ pancreatic cancer^12^. EV-packaged hnRNPA1 has been used to evaluate the occurrence of lymphovascular invasion and LN metastasis, and has shown excellent diagnostic performance, with a sensitivity exceeding 90%^12^. On this basis, we have proposed a combined panel of EV-packaged biomarkers for pancreatic cancer with different KRAS subtypes, to provide an alternative strategy for more accurate monitoring of LN metastasis in early stages.

Although EVs show promising prospects in the diagnosis of LN metastasis, open questions remain regarding their application. The basis of current understanding of the clinical relevance of EV-packaged molecules in LN metastasis has been restricted to single-center cohort studies or laboratory experiments. However, the applicability of EV-packaged molecules to diagnosis and treatment in clinical settings must be verified in multi-center clinical studies. In addition, the origin of EVs in body fluids is not homogeneous, and approaches for identification of EVs derived from cancer cells are expected to be developed. PBA provides a high-throughput method to detect the surface proteins of individual EVs. After binding of antibody-coupled oligonucleotide probes to RCA product molecules, RCA can be used to wrap EVs, so that surface proteins and antibody-coupled oligonucleotide probes are immunobound, thus converting information regarding EV surface proteins into oligonucleotide sequences. Individual EV surface protein expression profiles have been obtained through PCR amplification or RNA sequencing^18^. Moreover, because EVs reflect the characteristics of the original cells, detection at the single EV level might result in another major research advance in EV diagnosis.

Developing more efficient drugs targeting LN metastasis is another challenge. LN metastasis is an intricate and multi-step process whose mechanism has been explored in the past decade. A variety of targeted drugs have been investigated for blocking LN metastasis but have unfortunately shown quite limited results^1^, owing to low bioavailability, low intratumoral administration, rapid clearance, and systemic adverse effects^19^. To address these challenges, multiple nano-vehicles have been designed to improve theranostic capabilities and decrease clearance, among which EVs are considered with the most promising applications due to their mass production and intrinsic tumor-targeting abilities. Furthermore, substantial efforts have been made to identify the crucial contents and surface molecules, including lncRNAs or integrins, that contribute to the specific regulation of EVs during LN metastasis, to provide a basis for endowing EVs with metastatic niche-targeted properties by artificial engineered modification. Engineering membrane proteins on interstitial cells, such as generating interleukin-2-tethered T cells to produce IL-2-positive EVs, improves anti-cancer efficacy by targeting immune checkpoints, thus enhancing the clinical applicability of immunotherapeutic agents^20^. However, substantial areas remain to be addressed in the development of EV delivery systems targeting the specific steps of LN metastasis. Recent studies have shown that interfering with RNA components in EVs to inhibit their effects on lymphangiogenesis significantly decreases the occurrence of LN metastasis in mouse models^9,11^. In addition, we have recently shown that the regulatory downstream targets of KRAS mutation trigger EV production in pancreatic cancers and facilitate LN metastasis in a VEGF-C-independent manner^9,11^. EV membrane proteins recognize cell membrane surface receptors in the TME and transport molecules between cells. On the basis of linking LAMP-2B with the CDS region sequence of the target protein, EVs have been collected for identification and targeting verification, to screen protein-binding peptides targeting specific cells or tissues. The engineering of EVs containing targeted peptides provides an effective drug delivery tool for targeted therapy to block tumor lymphatic metastasis. Further analysis of the downstream pathways of KRAS mutation and the components of KRAS mutant responsive EVs is expected to identify the molecules regulating the targeting capacity of EVs. These molecules may be artificially loaded onto the surfaces of EVs to enhance the internalization of EVs by LECs. On this basis, the construction of EV delivery systems targeting lymphangiogenesis, alone or combined with anti-VEGF-C therapy, may provide an elegant strategy for blocking LN metastasis.
